# Degeneration Modulates Retinal Response to Transient Exogenous Oxidative Injury

**DOI:** 10.1371/journal.pone.0087751

**Published:** 2014-02-21

**Authors:** Michal Lederman, Shira Hagbi-Levi, Michelle Grunin, Alexey Obolensky, Eduard Berenshtein, Eyal Banin, Mordechai Chevion, Itay Chowers

**Affiliations:** 1 Department of Ophthalmology, Hadassah-Hebrew University Medical Center, and the Hebrew University-Hadassah School of Medicine, Jerusalem, Israel; 2 Department of Cellular Biochemistry and Human Genetics, Hadassah-Hebrew University Medical Center, and the Hebrew University-Hadassah School of Medicine, Jerusalem, Israel; University of Cologne, Germany

## Abstract

**Purpose:**

Oxidative injury is involved in retinal and macular degeneration. We aim to assess if retinal degeneration associated with genetic defect modulates the retinal threshold for encountering additional oxidative challenges.

**Methods:**

Retinal oxidative injury was induced in degenerating retinas (rd10) and in control mice (WT) by intravitreal injections of paraquat (PQ). Retinal function and structure was evaluated by electroretinogram (ERG) and histology, respectively. Oxidative injury was assessed by immunohistochemistry for 4-Hydroxy-2-nonenal (HNE), and by Thiobarbituric Acid Reactive Substances (TBARS) and protein carbonyl content (PCC) assays. Anti-oxidant mechanism was assessed by quantitative real time PCR (QPCR) for mRNA of antioxidant genes and genes related to iron metabolism, and by catalase activity assay.

**Results:**

Three days following PQ injections (1 µl of 0.25, 0.75, and 2 mM) the average ERG amplitudes decreased more in the WT mice compared with the rd10 mice. For example, following 2 mM PQ injection, ERG amplitudes reduced 1.84-fold more in WT compared with rd10 mice (p = 0.02). Injection of 4 mM PQ resulted in retinal destruction. Altered retina morphology associated with PQ was substantially more severe in WT eyes compared with rd10 eyes. Oxidative injury according to HNE staining and TBARS assay increased 1.3-fold and 2.1-fold more, respectively, in WT compared with rd10 mice. At baseline, prior to PQ injection, mRNA levels of antioxidant genes (*Superoxide Dismutase1, Glutathione Peroxidase1, Catalase*) and of *Transferrin* measured by quantitative PCR were 2.1–7.8-fold higher in rd10 compared with WT mice (p<0.01 each), and catalase activity was 1.7-fold higher in rd10 (p = 0.0006).

**Conclusions:**

This data suggests that degenerating rd10 retinas encounter a relatively lower degree of damage in response to oxidative injury compared with normal retinas. Constitutive up-regulation of the oxidative defense mechanism in degenerating retinas may confer such relative protection from oxidative injury.

## Introduction

Oxidative damage has been implicated in several neurodegenerative diseases among them Alzheimer’s [1], Huntington’s [2], Friedreich’s ataxia [3] and Parkinson’s [4]; as well as in pathologies affecting the retina such as retinitis pigmentosa [5], age related macular degene**r**ation (AMD) [6], [7] and glaucoma [8]. The retina is characterized by high oxygen and poly-unsaturated fatty acid content, and by light exposure, which render it vulnerable to oxidative injury [9]. Supplementation of zinc and antioxidants can slow the course of age related macular degeneration in humans [10], and retinal degeneration in mice, further implicating oxidative retinal injury in retinal and macular degeneration [11]–[14].

Retina and macula undergoing chronic oxidative injury during the course of genetically-driven degenerative processes might encounter additional transient exogenous oxidative stress stemming from exposure to a chemical generating reactive oxygen species (ROS), an inflammatory reaction, or light exposure. Limited data is available with respect to the threshold of the degenerating retina in comparison to the normal retina for such additional oxidative injury. If the degenerating retina is more susceptible to exogenous oxidative challenges then patients with retinal degenerations should be rigorously protected from oxidative challenges which are not harmful to the healthy retina.

We evaluated the extent of retinal damage following exposure to paraquat (PQ), a bipyridium herbicide that selectively generates superoxide radical anions, in mice with genetically–driven retinal degeneration (rd10 mice) and in C57BL/6 wild type (WT) mice [15], [16]. We then evaluated the anti oxidative enzymatic defense system and iron homeostasis to explore possible underlying causes for the different susceptibility to oxidative injury between rd10 and WT mice. Iron metabolism was characterized as it may contribute to oxidative injury in retinal and macular degeneration through the Fenton reaction [17], [18].

## Materials and Methods

### Mice

rd10 mice (on C57BL/6 background), homozygous for the missense mutation in the β-subunit of the rod phosphodiesterase gene (*Pde6b^rd10^*), were used as a model for retinal degeneration- accelerates around three weeks of age in this strain and after 60 days of age only few nuclei remain in the outer nuclear layer and the electroretinogram (ERG) is absent or barely recordable [16], [19]. C57BL/6 mice served as controls. Mice were maintained in a specific pathogen free animal facility, with a 12-hour light (white fluorescence, 30 cd/m^2^)/dark cycle, and an unlimited food and water supply. Animals were treated in accordance to the guidelines of the Association for Research in Vision and Ophthalmology, and experiments were conducted with the approval of the ethics committee of the Faculty of Medicine of the Hebrew University. Before all procedures mice were anesthetized by intraperitoneal injections of a mixture of ketamine (Bedford Laboratories, Bedford, OH) and xylazine (VMD, Arendonk, Belgium), with doses suitable to their body weight.

### Intravitreous Injections

Intravitreous injections were delivered using a PLI-100 Pico-Injector (Medical System Corp., Greenvale, NY), as previously described [20]. Three-week-old mice were anesthetized, and eyelids were drawn back. Under a dissecting microscope, a pulled glass micropipette was passed through the pars plana and mice were injected with 1 µl of 0.25, 0.75, 2 mM or 4 mM PQ (SIGMA-Aldrich, St. Louis, MO) diluted in phosphate buffered saline (PBS) (Biological Industries, Kibbutz Beit Haemek, Israel) in one eye. These concentrations were selected based on a previous report [15]. In that study, the 0.5 mM concentration did not cause retinal damage to wild type mice while 2 mM caused severe retinal injury. Contra-lateral eyes, injected with 1 µl of PBS, served as controls.

### Electroretinograms (ERG)

Three days following intraocular injections, full field ERGs were recorded in anesthetized mice following over-night dark adaptation. Pupils were dilated with 1% tropicamide and 2.5% phenylephrine, and benoxinate HCl 0.4% (all from Fisher Pharmaceuticals, Tel-Aviv, Israel) drops were applied to the corneas as local anesthetics prior to gold-wire active electrode placement on the central cornea. A reference electrode was placed in the tongue and a needle ground electrode was placed intramuscularly in the hip area. ERGs were recorded inside a Faraday cage using the Espion computerized system (Diagnosys Llc, Littleton, MA). Dark adapted ERG responses to a series of white flashes of increasing intensities (from 0.000006 to 9.6 cd*sec/m^2^) were recorded with inter-stimulus intervals rising from 10 sec for lowest intensity flashes to 90 sec for highest intensity flashes. Light adaptation was accomplished with a background illumination of 30 cd/m^2^. Cone 1 Hz and 16 Hz flicker ERGs to a series of white flashes (from 0.34 to 9.6 cd*sec/m^2^) were recorded. All responses were filtered using 0.3 to 500 Hz filters and signal averaging was applied [21]. Amplitudes of a- and b-waves were then measured and analyzed.

### Tissue Processing

Mice were euthanized with an overdose of ketamine, followed by cervical dislocation. For frozen sections (used for immunostaining), five-day post-injection of PQ eyes were enucleated and immediately placed in Davidson solution [25 ml Glacial Acetic acid, 71.25 ml Ethanol (BIO LAB, Jerusalem, Israel), 50 ml 10% neutral buffered formalin (EMS, Hatfield, PA), and 78.75 ml double distilled water] for overnight fixation. The following day, eyes were transferred to 30% sucrose for 24 hours, after which they were frozen in blocks of Tissue-Tek optimum cutting temperature (O.C.T, Sakura, Torrance, CA) embedding compound, and frozen on dry ice. Eyes were sliced into 6 µm thick sections, along the corneal-optic nerve axis, using a Leica CM 1100 cryostat (Heidelberger, Germany). Conventional histology was performed on formalin-fixed, paraffin-embedded sections using hematoxylin and eosin staining.

For all other procedures retinas were gently separated from freshly enucleated eyes under a dissecting microscope, immediately frozen in liquid nitrogen and stored at −80°C until further use.

### Immunohistochemistry (IHC)

Immunostaining was performed as we have recently described [17]. Briefly, immunostaining with rabbit anti 4-hydroxy-2-nonenal (HNE; diluted 1∶100 in 1% BSA; Alpha Diagnostics, San Antonio, TX) was done to assess the extent of oxidative damage. Cy3-conjugated goat anti rabbit antibody (diluted 1∶200; Jackson ImmunoResearch, West Grove, PA), was used as secondary antibody and slides were counterstained with DAPI (Santa Cruz Biotechnology, Santa Cruz, CA) [22]. Sections were viewed through fluorescent microscopy (Olympus BX41, Tokyo, Japan), using appropriate filters. Background was controlled by setting the exposure parameters as such that provide no detectable signal for the control section and these same parameters were maintained while capturing images from the test sections. Images were photographed with an Olympus DP70 digital camera.

Quantification of immunochemistry was performed on digital images using ImageJ Software (http://rsb.info.nih.gov/ij/index.html) [23] by measuring the average staining intensity per pixel in five replicas of sections chosen from each sample at a similar distance from the optic nerve. In each section, staining was measured in an area including the entire thickness of the retina from the inner limiting membrane to the RPE (retinal pigment epithelium).

### Quantitative Real-Time RT-PCR (QPCR)

Total RNA was extracted from a pool of two flash-frozen retinas (indicated as paired) using TRI Reagent (SIGMA), according to the manufacturer’s instructions, and then treated with DNAase (TURBO DNA-free, Ambion, Austin, TX). Reverse transcriptase polymerase chain reaction was performed using the High Capacity cDNA Reverse Transcription Kits (Applied Biosystems, Foster City, CA) and anchored oligo dT primers on 1 µg of RNA. QPCR was performed to measure mRNA levels of genes involved in iron metabolism and of anti-oxidant enzymes. Measurement of the anti-oxidant enzymes mRNA levels was performed on retinas prior to injection (4 pairs of WT retinas-8 retinas total, and 7 pairs of rd10 retinas- 14 retinas total), while measurement of the genes involved in iron metabolism was performed on retinas five days after injection with either a PBS control or injection of the contralateral eye with 1 µl of 2 mM PQ. Six pairs (12 retinas total) of rd10 eyes and six pairs of WT eyes were injected with PBS, while six pairs (12 retinas total) of contralateral rd10 or WT eyes were injected with PQ. Measurement of *GAPDH* and *HPRT* mRNA levels served as endogenous controls. All reactions were carried out in triplicate at a total volume of 20 µl. Wells contained 40 ng cDNA template, 0.75 µl TaqMan Gene Expression assay [*glyceraldehyde-3-phosphate dehydrogenase (GAPDH)*: Mm99999915_g1; *hypoxanthine guanine phosphoribosyl transferase (HPRT):* Mm00446968_m1; *catalase (CAT):* Mm00437992_m1; *superoxide dismutase 1 (SOD1):* Mm01344233_g1; *glutathione peroxidase 1 (GPX1):* Mm00656767_g1; *ceruloplasmin (Cp)*: Mm00432654_m1; *transferrin (Tf)*: Mm01230431_m1; *transferrin receptor (Tfrc)*: Mm00441941_m1], 7.5 µl TaqMan Universal PCR Master Mix (Applied Biosystems) and were completed with double distilled water, and standard TaqMan technique was applied. Using 96 well plates, signal amplification was measured throughout 45 cycles of 60°C for 15 seconds, followed by 95°C for 15 seconds. Fluorescent signals were measured by the system StepOnePlus (Applied Biosystems) and analyzed using the Step One Software version 2.2 to obtain threshold cycle (CT) values (Applied Biosystems), along with spreadsheet software (Excel; Microsoft, Redmond, WA). Expression levels of each gene were compared by using the geometric mean of the endogenous controls [24] according to the standard 2^(**-**ΔΔCT)^ calculation [25], giving results as relative quantification and fold change ± standard error of the mean (SEM**)**.

### Protein Isolation

Lysis buffer containing 1% deionized TritonX-100 and 0.1% sodium azide in 50 mM Tris-HCl (SIGMA), pH 7.5, was incubated with Chelex-100 (Bio-Rad, Hercules, CA), for 24 hours. Immediately before use, 0.25 mM Phenyl-Methyl-Sulfonyl-Fluoride was added (1∶1000, SIGMA). Pools of retinal tissue were homogenized in the buffer, sonicated at 10 watts for 1 min, and stored on ice for half an hour while vortexing every 5 minutes. Samples were then centrifuged at 2,750 *g*, for 15 min, at 4°C, after which supernatant was separated and refrozen at −80°C. Protein content was estimated using the BCA Protein Assay Kit (Pierce, Rockford, IL). Ferritin levels were measured by ELISA as was previously described [17], [26].

### Thiobarbituric Acid Reactive Substances (TBARS) Measurement

Lipid peroxidation was evaluated by measuring levels of MDA (Malonaldehyde-bis-DimethylAcetal) [27]. Eight retinas were homogenized in 800 µl lysis buffer (see above) and centrifuged at 10,000 *g* for 10 min at 4°C. 200 µl of the supernatant were added to 100 µl 8.1% SDS (BDH Chemicals, Poole, UK), 750 µl 20% acetic acid (Frutarom, Haifa, Israel) pH 3.5, and 750 µl 0.8% thiobarbituric acid (SIGMA), and incubated for half an hour at 100°C. After cooling, 1.35 ml n-butanol:pyridine (15∶1; BIO LAB:SIGMA) were added, and the mixture was centrifuged at 4,000 *g* for 15 min at 4°C. Absorbance of the organic phase was measured using a UVKON_XL_ spectrophotometer (Bio-Tek, Winooski, VT) at λ = 532 nm. The amount of TBARS was determined according to a standard calibration curve generated from MDA.

### Protein Carbonylation

Protein carbonyl content (PCC) was quantified as an indicator for protein damage in the retina. Samples and controls were each measured in duplicates. Supernatant (100 µl, prepared as described above for TBARS analysis) was reacted with 400 µl 10 mM 2,4-dinitrophenylhydrazine (DNPH, Aldrich) in 2M HCl (Frutarom), or 2M HCl alone (for controls). Reaction tubes were incubated for one hour at room temperature in the dark, while vortexing every 15 min. 500 µl 20% tri chloro-acetic acid (TCA, SIGMA) were added, followed by five min incubation on ice, after which tubes were centrifuged at 10,000 *g* for 10 min at 4°C. Supernatant was discarded; pellets were re-suspended in 1 ml 10% TCA, and again incubated and centrifuged as described above. Pellets were washed three times with ethanol:ethylacetate (1∶1, SIGMA) for 10 min, and centrifuged after each wash. The pellets were re-suspended in 500 µl 6 M guanidine-hydrochloride (BIO LAB) in 0.5 M K_3_PO_4_ (SIGMA) pH 2.5, and again centrifuged. Absorbance was measured using a spectrophotometer at λ = 370 nm. Protein carbonyl content was established using the corrected absorbance (CA) that was calculated by subtracting the average absorbance of the controls from that of the samples [28]–[30].

### Catalase Activity

Catalase activity was evaluated by measuring the formaldehyde produced by reacting the samples with methanol in the presence of H_2_O_2_, as previously described [31]. Briefly, pairs of retinas were combined and homogenized in 500 µl cold 25 mM KH_2_PO_4_ (Riedel-de Haen, Hanover, Germany) pH 7 buffer, centrifuged at 10,000 *g* for 15 min at 4°C, and stored on ice. 100 µl of the supernatant were transferred into a tube containing 50 µl buffer and 50 µl 100% methanol (BIO LAB). The reaction was initiated by adding 10 µl 0.27% H_2_O_2_ (BIO LAB). Samples were incubated for 20 min at room temperature on a shaker. 50 µl 7.8 M KOH (Frutarom) was added to terminate the reaction. 100 µl 34.2 mM purpald (SIGMA) in 480 mM HCl were then added and tubes were again placed on a shaker for 10 min. The perpald was then oxidized by adding 50 µl 65.2 mM potassium periodate (Alfa Aesar, Karlsruhe, Germany) in 470 mM KOH, resulting in the formation of a purple colored product which, after spinning at 9,500 *g* for 10 min, was quantified by measuring absorption at λ = 550 nm. Amounts of formaldehyde formed were determined by generating a standard calibration curve. One unit of enzymatic activity is defined as the amount of catalase necessary to cause the formation of one nmol formaldehyde per minute at room temperature.

### Statistical Analysis

All data are presented as means ± SEM. For ERG analysis statistical significance was calculated using the two tailed paired *t*-test with Welch correction, when results were of normal distribution, otherwise non-parametric Wilcoxon matched pairs test was performed. Significance of HNE immunostaining and TBARS assay was calculated using two-way ANOVA. The student's *t*-test or Mann-Whitney test was applied for all other calculations. Statistical calculations were performed using InStat software (GraphPad Software, La Jolla, CA). Differences were considered significant at p-value <0.05.

## Results

### Retinal Function and Structure Following Intravitreal PQ Injection

Three-week-old C57BL/6 (wild type, WT) and rd10 mice received intravitreal injections of 1 µl of 0.25, 0.75, 2 or 4 mM PQ in one eye, and 1 µl PBS in the fellow eye (n = 10–22 in each group). Three days post injection full field ERGs were recorded to assess retinal function.

**Figure 1:**
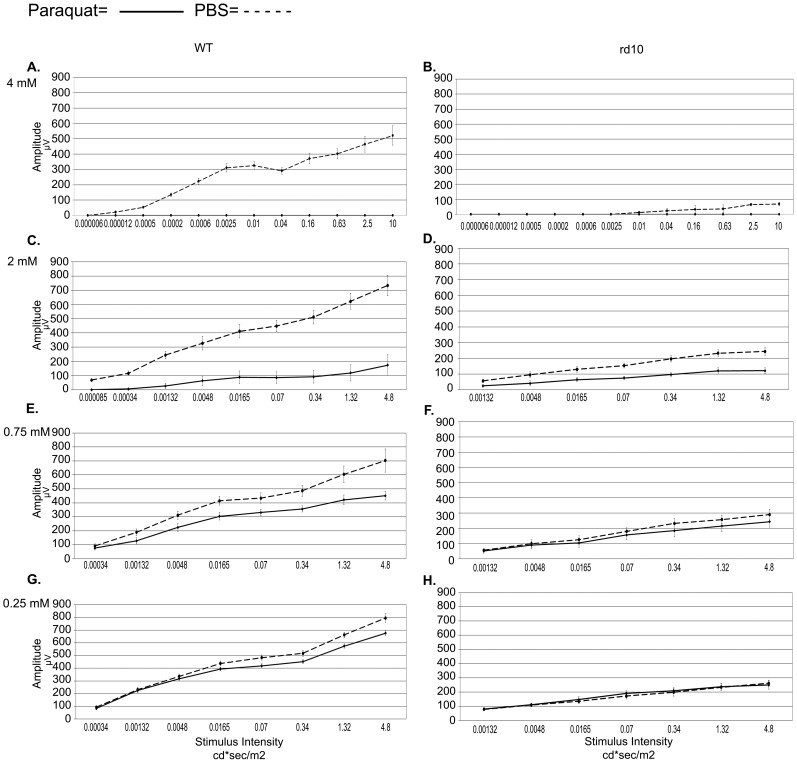
Scotopic b-wave ERG responses following intravitreal injection of PQ. ERGs were recorded three days following injection of 4(A,B), 2 mM (C,D), 0.75 mM (E,F), or 0.25 mM (G,H) of PQ in one eye and PBS in the fellow eye of wild type (WT) and rd10 mice (n = 10–22 in each group). Y-axis shows amplitude in microvolt while the x-axis shows the stimulus intensity.

**Figure 2:**
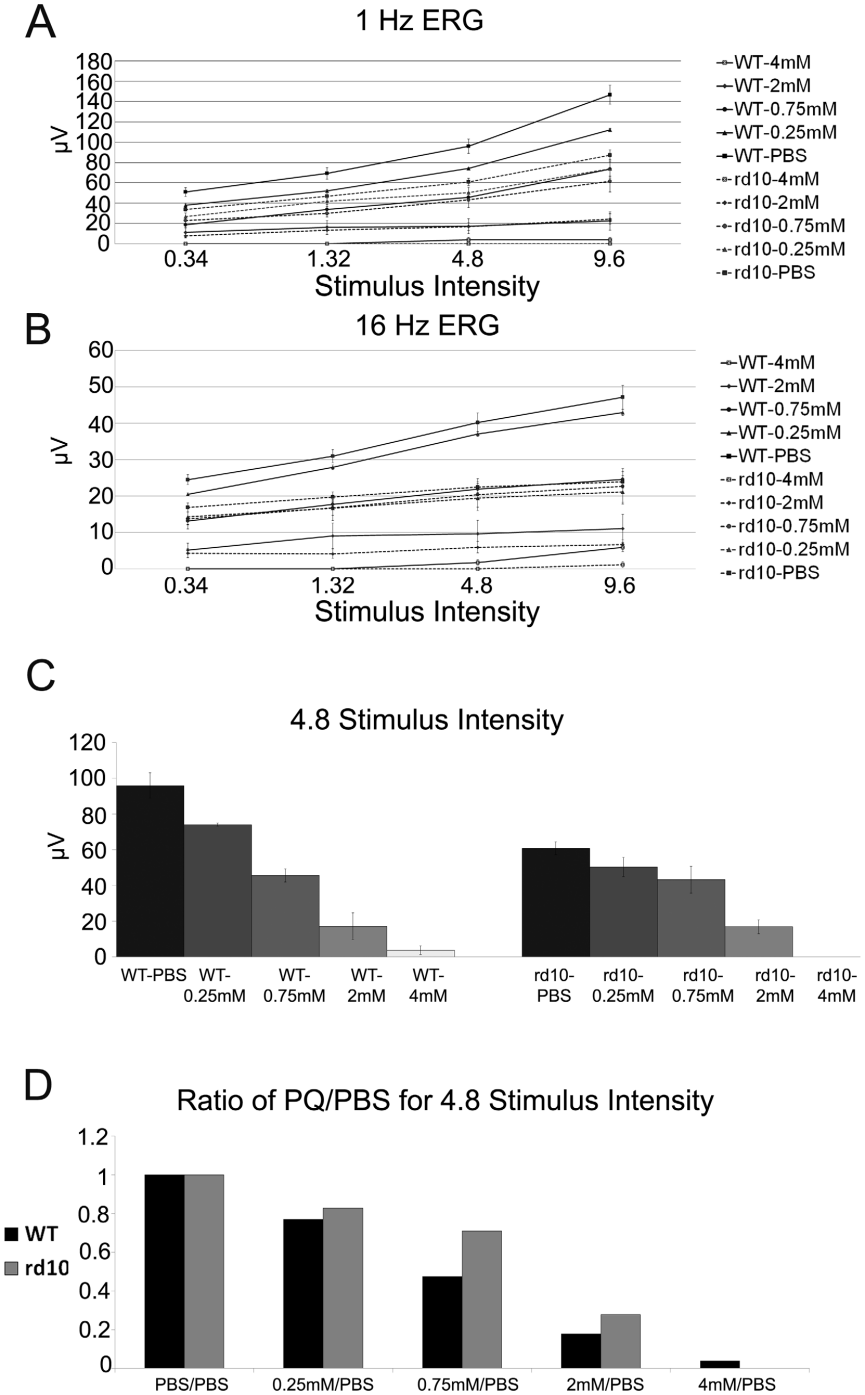
Photopic 1 Hz and 16 Hz ERG amplitudes in wild type (WT) and rd10 mice following PQ or PBS injection. (A) 1 Hz photopic amplitude shows both WT and rd10 mice after PQ or PBS injection. (B) indicates 16 Hz ERG amplitude for same WT and rd10 mice after PQ or PBS injection. 1 Hz b-wave amplitudes for each experimental group at 4.8 stimulus intensity are displayed in panel C. Average b-wave amplitudes at highest signal intensity divided by b-wave amplitudes from PBS injected eyes for each mouse group at each of the PQ concentrations is summarized in panel D. Larger reduction in ERG amplitudes following PQ injection was observed in WT compared with rd10 mice (please see result section for details). n = 10–22 mice in each group.

As expected, in rd10 mice treated by intravitreal injections of PBS, ERG amplitudes were significantly lower compared to the wild type eyes. A dose-dependent response to PQ injection was observed in both rd10 and WT mice (Fig. 1A–H Fig 2 A–D and Figure S1). Yet, WT mice manifested a more prominent reduction in ERG responses compared with the response of rd10 mice to PQ injection (Fig. 1&2). For example, following 2 mM PQ injection the average scotopic b-wave amplitude at maximal stimulus intensity in WT mice dropped by 79% while in rd10 mice the drop was 47% (p = 0.02). Furthermore, injection of 0.25 mM PQ did not affect the ERG responses of rd10 mice while it significantly reduced responses were recorded in WT mice (Fig. 1). The average scotopic b-wave amplitude at maximal stimulus intensity in WT eyes injected with 0.25 mM PQ was 676±21 µV while in control eyes was 795±34 µV (p = 0.009). Light adapted responses to 1-Hz and 16-Hz stimuli corroborated recordings from dark adapted eyes (Fig. 2). The larger relative decline in ERG recordings from WT mice compared with rd10 mice following PQ injection was highlighted by plotting the ratio of photopic 1 Hz ERG amplitudes at increased concentrations of paraquat injections vs. PBS injection (Fig. 2D). Larger drop in ERG recording is evident in response to injection of 0.25, 0.75, and 2 mM, while injection of 4 mM resulted in flattening of the signal from the rd10 retinas.

**Figure 3:**
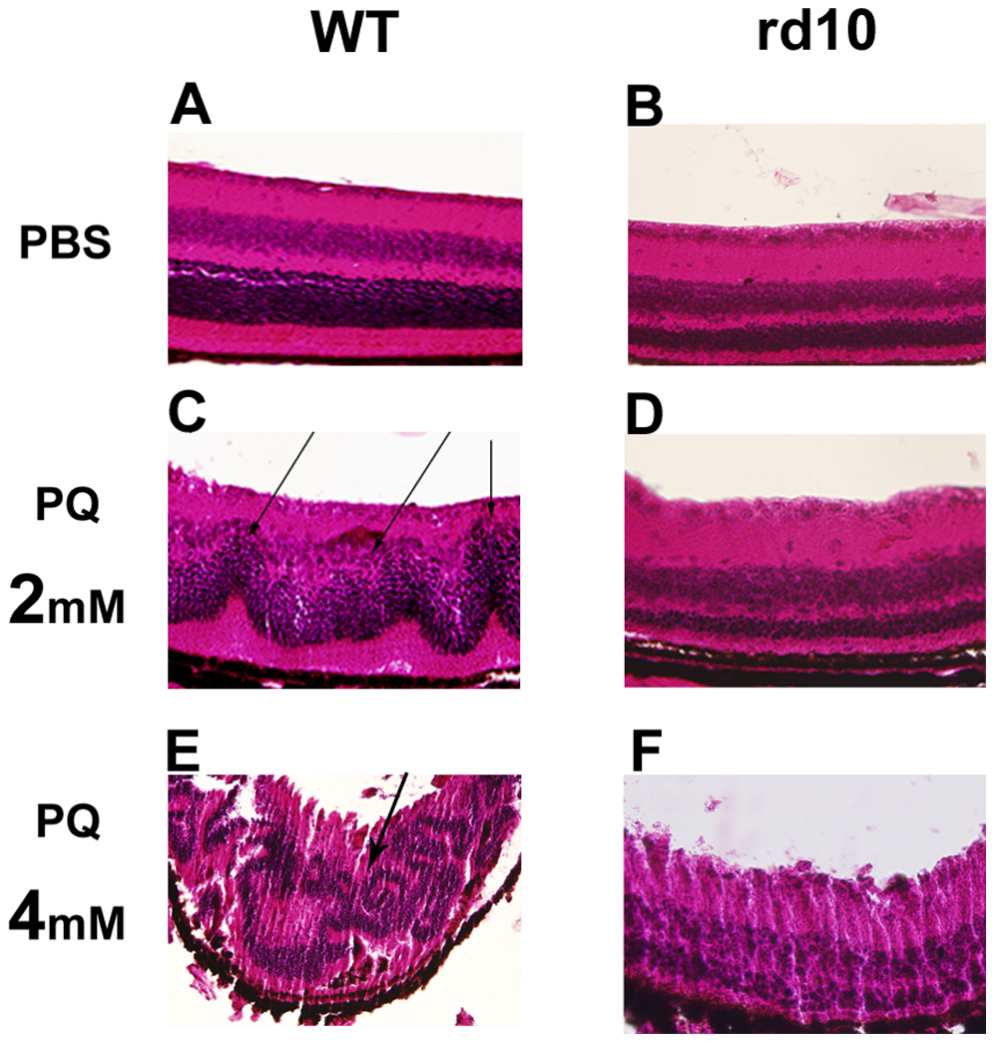
H&E staining of formalin-fixed paraffin-embedded retina sections from rd10 and WT mice injected with 1 µl of 2 mM or 4 mM PQ or PBS. Normal retina morphology was seen in WT mice (A), while the retina appeared disrupted in these mice following PQ injection (C, E). Arrow indicates wavy appearance of nuclear layers and photoreceptor inner and outer segments in WT retina injected with PQ. This wavy appearance appear extreme following injection of 4 mM PQ. Retinas of rd10 mice, already undergoing retinal degeneration, are typically thinner, due to loss of photoreceptors (B). PQ injection in rd10 mice was not associated with structural alterations similar to the one observed in WT mice (D, F). GCL =  ganglion cell layer, INL = inner nuclear layer, ONL = outer nuclear layer, RPE =  retinal pigmented epithelium.

Distorted outer nuclear layer (ONL) morphology was evident in sections from 9 of the 11 of the WT mice treated by 2 mM of PQ which were evaluated. By contrast, rd10 mice retinas showed ONL thinning secondary to the degenerative process, but mild distorted morphology was identified only in one eye of the 11 which were evaluated (P = 0.0019; Fig. 3). Similarly, injections of 4 mM PQ resulted in massive alterations in retinal structure in WT retinas while more subtle alterations were detected in rd10 retinas (Fig. 3). Exact quantification of the ONL was hampered by the complete disorganization of the retinal layers due to PQ-associated damage.

### Oxidative Retinal Injury

**Figure 4:**
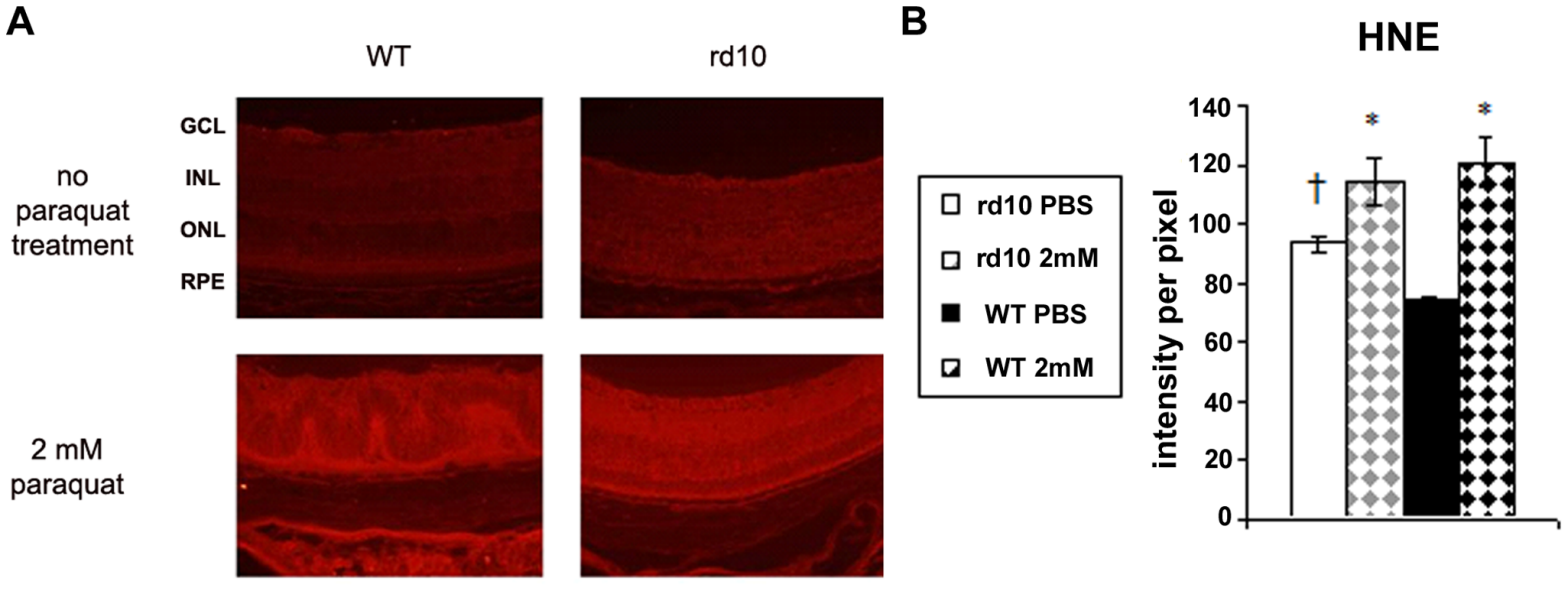
HNE staining of retina sections for assessment of oxidative injury following PQ injection. Retinas of rd10 and WT mice injected with 1 µl of 2 mM PQ or PBS were labeled with anti-HNE antibody (*red*; A). GCL =  ganglion cell layer, INL = inner nuclear layer, ONL = outer nuclear layer, RPE =  retinal pigmented epithelium. (B) Quantification of HNE staining intensity showed marked oxidative injury following PQ injection in WT and the rd10 mice (*p<0.05 as compared to PBS injected eyes of same strain. † p = 0.0002 comparing control (PBS) eyes between the strains; n = 5 in each group).

HNE staining and TBARS assay were employed as markers for oxidative injury to fatty acids. Immunostaining for HNE was performed on retinal sections of rd10 and WT mice injected with 2 mM PQ or PBS. Results showed staining in all retinal layers (Fig. 4A). In PBS injected eyes, HNE staining was 1.3-fold higher in rd10 compared with WT retinas (p = 0.0002). Following PQ injection HNE staining intensity increased by 1.2-fold (p = 0.04) in rd10 mice and by 1.6-fold (p = 0.0003) in WT mice. This difference in the fold-increase of HNE staining following PQ injection between WT and rd10 mice was significant (p = 0.04; Fig. 4B).

**Figure 5:**
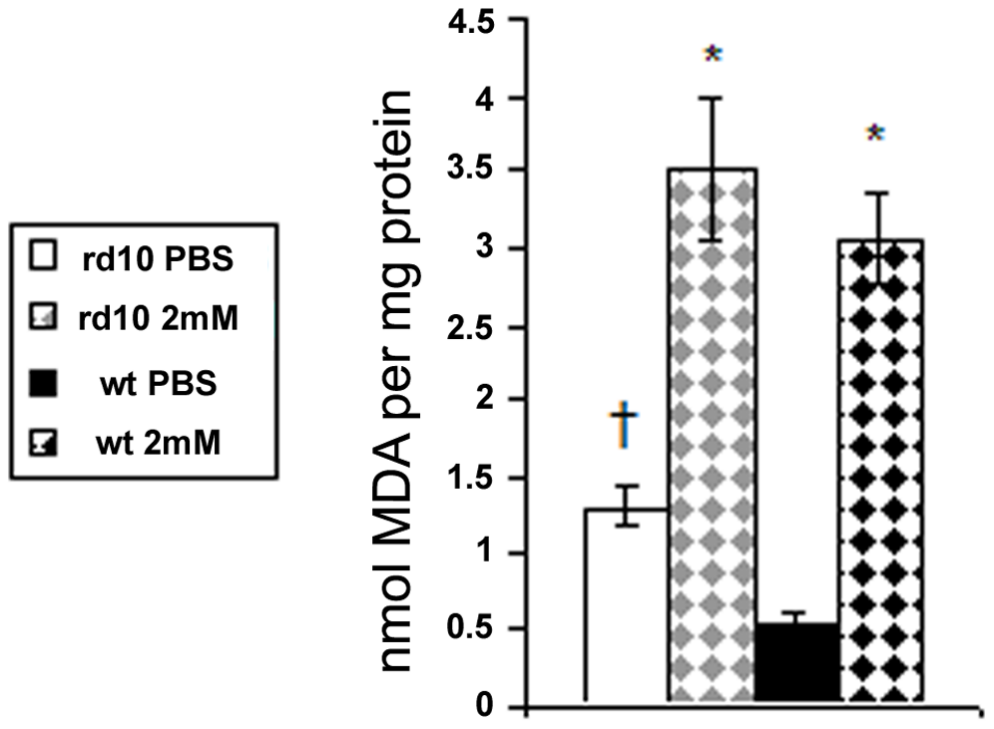
Measurements of TBARS level in mice retinas following PQ injection. TBARS level (nmol MDA per mg protein) indicates the extent of oxidative injury to lipids. TBARS were measured in retinas of rd10 and WT mice following 2 mM paraquat or PBS injections. *p≤0.004 as compared to PBS injected eyes of same strain. † p = 0.001 comparing control (PBS) eyes between the strains (n = 5 in each group).

TBARS levels were 2.4-fold higher in PBS injected rd10 eyes compared to PBS injected C57BL/6 eyes (p = 0.001). Following PQ injection there was a 2.7-fold and 5.7-fold rise in TBARS levels in rd10 (p = 0.004) and WT (p<0.0001) retinas compared to controls, respectively. This difference in the fold-increase of TBARS following PQ injection between WT and rd10 mice was not significant (p = 0.6; Fig. 5).

**Figure 6:**
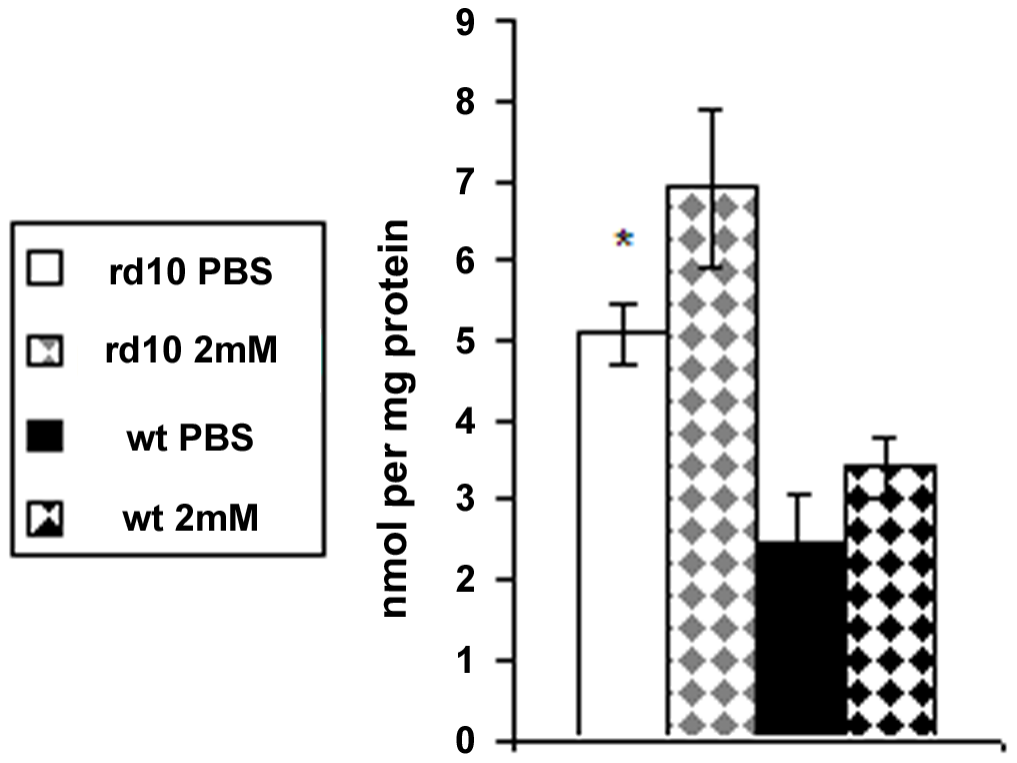
Protein carbonyl content (PCC) in mice retinas following PQ injection. Protein carbonylation was measured to assess oxidative retinal injury to proteins. It was assessed in retinas of rd10 and WT mice injected with 1 µl of 2 mM PQ or PBS. In both strains PQ injections caused an insignificant 1.35 fold increase of PCC. *p = 0.009 comparing control (PBS) rd10 with WT retinas (n = 5 in each group).

Oxidative injury to retinal proteins was evaluated by measurement of protein carbonyl content (PCC) in control and 2 mM PQ injected eyes. The PCC measured was 2.1-fold higher in control rd10 retinas compared with WT retinas (p = 0.009; Fig. 6). Both strains showed a 1.3-fold rise in PCC in PQ injected eyes compared with control eyes.

### Antioxidant Levels Following Intravitreal PQ Injection

**Figure 7:**
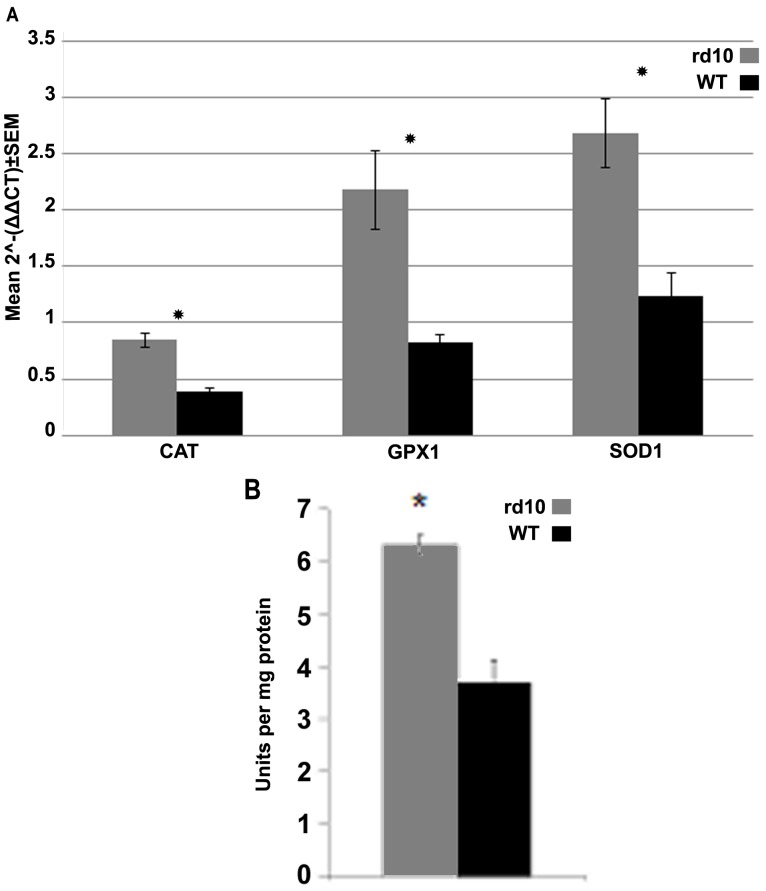
Retinal Expression of antioxidant genes in rd10 and WT mice. (A) mRNA levels of anti-oxidant enzymes in normal and degenerating retinas. *SOD1*, *GPX1,* and *CAT* expression levels were measured using QPCR (rd10 n = 7 pairs, WT n = 4 pairs). (B) Catalase activity evaluated in retinas of three-week-old WT and rd10 mice. *p<0.05 in comparison to WT (n = 5 in each group).

To obtain insight into factors underlying the smaller effect of PQ on retinas from rd10 versus WT mice, we examined the mRNA levels of anti-oxidant enzymes involved in the removal of superoxide radical anion – superoxide dismutase (SOD1), and removal of hydrogen peroxide – catalase (CAT) and glutathione peroxidase (GPX1). mRNA levels of all three genes measured in retinas of three-week-old mice before injection were significantly higher in rd10 mice compared to the WT: *SOD1*- 2.17-fold (p = 0.04), *GPX1*–2.65-fold (p = 0.01) and *CAT* ’.1-fold (p = 0.02) (Fig. 7A).

QPCR results were confirmed by assessing catalase enzymatic activity. Results corroborated those of the expression assay, demonstrating a 1.7-fold increase in catalase activity in rd10 mice compared to the WT (p = 0.0006) (Fig. 7B).

### Assessment of Iron Metabolism

**Figure 8:**
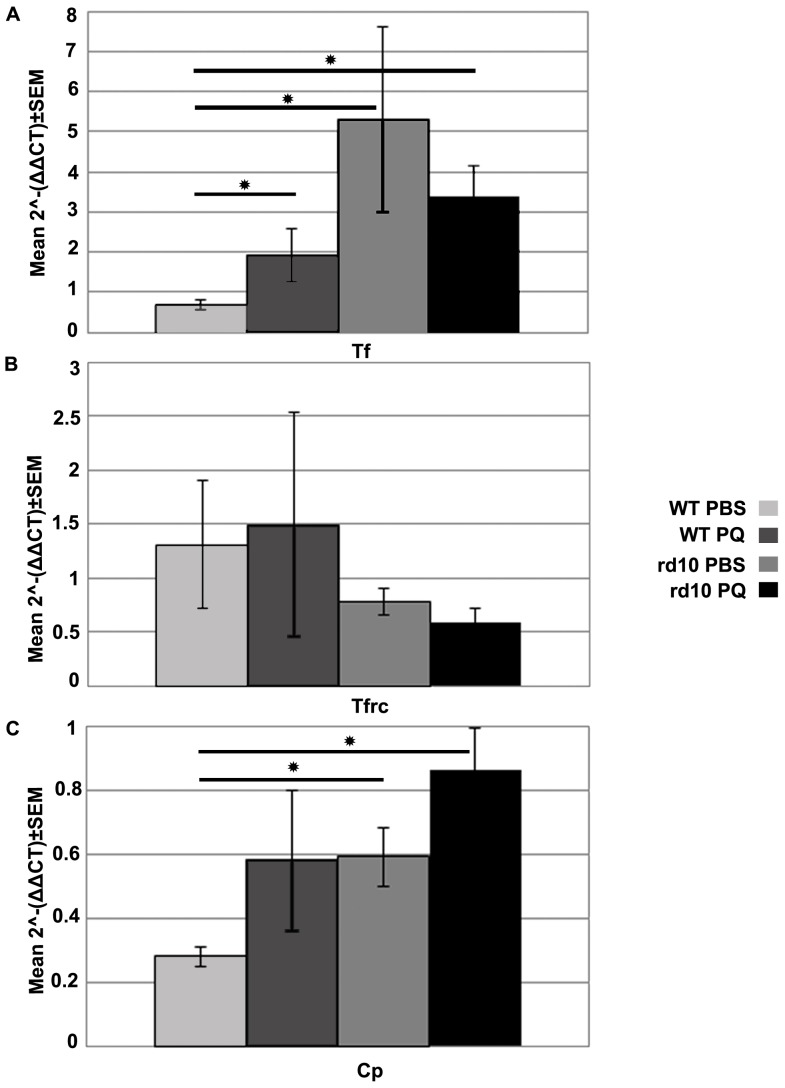
mRNA levels of iron metabolism associated genes. (A) *transferrin (Tf)* expression, (B) *transferrin receptor* (*Tfrc)* expression, (C) *ceruloplasmin (Cp)* expression, measured in rd10 and WT retinas following intravitreal injection of 1 µl of 2 mM PQ or PBS. Bars = mean relative mRNA levels ± SEM. *p<0.05 as compared to PBS injected eyes (PBS) of same strain. † p<0.05 comparing expression in control eyes between the strains (n = 6 pairs in each group).

Alterations in iron homeostasis were evaluated by measurement of mRNA levels of the genes: *transferrin (Tf), transferrin receptor (Tfrc), and ceruloplasmin (Cp),* along with quantification of ferritin protein level. *Tf* mRNA levels were higher in PBS-injected rd10 eyes (7.8-fold, p<0.0001), PQ-injected rd10 eyes (5-fold, p = 0.0003), and PQ-injected WT eyes (2.8-fold, p = 0.03) compared with PBS-injected WT eyes, respectively (Figure 8A).

mRNA levels of *Tfrc* were not affected by PQ or PBS injection in WT or rd10 retinas (Figure 8B). *Cp* mRNA levels were lower in PBS-injected WT retinas compared with PBS-injected rd10 retinas (2.1-fold, p = 0.082) and PQ-injected rd10 retinas (3.07-fold, p = 0.0017; Figure 8C).

Ferritin protein levels were measured by ELISA in both types of mice after intravitreal injection of 2 mM PQ or PBS. Compared to WT eyes, rd10 mice showed a trend toward higher ferritin levels (1.5-fold, p = 0.07). PQ injection caused a slight rise in mean ferritin content in both strains, however no significant changes were observed (data not shown).

## Discussion

PQ injection to the vitreous cavity results in a dose dependent oxidative retinal injury [15], [16], [32]. In this study we have performed intravitreal injections of four different concentrations of PQ to assess the susceptibility of the degenerating retina for encountering exogenous oxidative stress. The results showed that relative to the pre-injection condition of the retina, rd10 mice suffered less retinal damage following PQ injection compared with WT mice. This relative resilience to PQ-associated damage in rd10 mice manifested in ERG responses, histology, and extent of oxidative injury to lipids as measured by HNE immunostaining and TBARS assay. The relative resilient of the rd10 retinas to PQ injury was overcome by the 4 mM concentration which resulted in total retinal destruction according to ERG. This last finding suggests that a floor effect did not underline the relative resistance of the rd10 retinas compared with WT retinas which was observed in the 0.25, 0.75, and 2 mM PQ concentrations.

Interestingly, at the time point which we have evaluated retinal lipids seemed to suffer the brunt of the injury, as both assays of lipid peroxidation showed substantial increase in lipid peroxidation following PQ injections, while protein carbonylation did not change significantly. These findings are in agreement with those of Cingolani *et al*. who demonstrated that protein carbonyl adducts were elevated five days but not three days following intravitreal PQ injections to WT eyes [15]. In that study PQ injections to WT retinas caused superoxide radical levels to rise, resulting in apoptosis and thinning of the inner and outer nuclear layers.

To gain insight into the factors that underlie the relative limited retinal damage in rd10 mice compared with WT mice following PQ injection we evaluated the oxidative defense and iron metabolism systems. Prior to PQ injection there were increased mRNA levels for three anti-oxidative enzymes including superoxide dismutase 1, gluthatione peroxidase 1, and catalase in rd10 mice retinas compared with WT retinas. The functional significance of this increased expression was validated by demonstration of increased catalase activity in rd10 retinas. This data suggests that in retinas undergoing chronic oxidative stress there is up-regulation of the oxidative defense mechanism that may conceivably confer partial protection from transient oxidative challenges. Our results correlate with those of Usui *et al.* who showed that co-expression of *CAT* and *SOD2*, and co-expression of GPX and SOD1 cause a reduction in oxidative damage and delay cone degeneration in rd10 mice [33], [34]. Similarly, Dong *et al.* showed that mice deficient in the *SOD1* gene are more sensitive to PQ injury to the retina, hence, identifying this gene as an important component of the defense system of the retina against this type of challenge [35].

Altered iron homeostasis and iron overload were implicated in retinal and macular degeneration, including in rd10 mice, and it was suggested that iron may exacerbate oxidative retinal injury in these diseases by generation of ROS through the Fenton reaction [13], [17], [21], [36]–[38]. In addition, Chen *et al.* found alterations in levels of iron metabolism associated proteins in normal aged rodent retinas along with elevated retinal iron. *In-vitro* studies showed that excess iron increased the susceptibility of retinal neurons to PQ toxicity, leading the authors to conclude that iron may contribute to the progression of age-related neurodegenerative diseases [39].

In the present study we have found increased *transferrin* mRNA levels and ferritin protein levels in rd10 mice compared with controls. Following PQ injection, levels of *Tf* increased in WT mice, while *ceruloplasmin* levels increased in WT and rd10 mice. This data suggests that altered retinal iron metabolism which was reported in the context of genetically driven retinal degeneration [17], [20], [22], [27], [37], photic retinal injury in mice [40], [41], ageing in rodents [39], and in age related macular degeneration in humans [18], [37], also occurs following exposure to oxidative stress.

We previously reported and further validated in the present study that transferrin and ferritin are up-regulated in rd10 mice retinas. Transferrin, an iron transporter, and ferritin, the major intracellular iron storage molecule, may decrease the availability of iron to the Fenton reaction, thereby, ameliorating oxidative injury. This possibility is supported by the fact that ferritin binding sites in the rd10 retina are not saturated [17], as well as by amelioration of retinal injury following intra peritoneal transferrin supplementation in rd10 mice [42].

Additional studies are required to evaluate if our findings may be extended to other sources of oxidative injury such as photic damage and hyperoxia or hypoxia. It would be of particular importance to clarify if transient exposure to environmental sources of oxidative injury, such as light, is harmful to the degenerating human retina in diseases such as macular degeneration and retinitis pigmentosa, and if treatment with anti-oxidants may alter the threshold of the human retina to encounter such stress.

## Supporting Information

Figure S1 **Traces of ERG recordings from WT (C57Bl6) and rd10 mice.** The graph shows an example of measurements of b-wave response under scotopic and photopic conditions as well as measurements of 16 Hz flicker ERG response. Cursor position for measurement of the b-wave is indicated.
(TIF)

## References

[1] Zhu X, Su B, Wang X, Smith MA, Perry G (2007) Causes of oxidative stress in Alzheimer disease. Cell Mol Life Sci 64: 2202–2210. 17605000 10.1007/s00018-007-7218-4 PMC11136009

[2] Trushina E, McMurray CT (2007) Oxidative stress and mitochondrial dysfunction in neurodegenerative diseases. Neuroscience 145: 1233–1248. 17303344 10.1016/j.neuroscience.2006.10.056

[3] Calabrese V, Lodi R, Tonon C, D’Agata V, Sapienza M, et al (2005) Oxidative stress, mitochondrial dysfunction and cellular stress response in Friedreich’s ataxia. J Neurol Sci 233: 145–162. 15896810 10.1016/j.jns.2005.03.012

[4] Jenner P (2003) Oxidative stress in Parkinson’s disease. Ann Neurol 53 Suppl 3S26–36 discussion S36–28. 12666096 10.1002/ana.10483

[5] Shen J, Yang X, Dong A, Petters RM, Peng YW, et al (2005) Oxidative damage is a potential cause of cone cell death in retinitis pigmentosa. J Cell Physiol 203: 457–464. 15744744 10.1002/jcp.20346

[6] Beatty S, Koh H, Phil M, Henson D, Boulton M (2000) The role of oxidative stress in the pathogenesis of age-related macular degeneration. Surv Ophthalmol 45: 115–134. 11033038 10.1016/s0039-6257(00)00140-5

[7] Shen JK, Dong A, Hackett SF, Bell WR, Green WR, et al (2007) Oxidative damage in age-related macular degeneration. Histol Histopathol 22: 1301–1308. 17701910 10.14670/HH-22.1301

[8] Sacca SC, Izzotti A, Rossi P, Traverso C (2007) Glaucomatous outflow pathway and oxidative stress. Exp Eye Res 84: 389–399. 17196589 10.1016/j.exer.2006.10.008

[9] Anderson RE, Kretzer FL, Rapp LM (1994) Free radicals and ocular disease. Adv Exp Med Biol 366: 73–86. 7771292 10.1007/978-1-4615-1833-4_6

[10] Age-Related Eye Disease Study Research Group (2001) A randomized, placebo-controlled, clinical trial of high-dose supplementation with vitamins C and E, beta carotene, and zinc for age-related macular degeneration and vision loss: AREDS report no. 8. Arch Ophthalmol 119: 1417–1436. 11594942 10.1001/archopht.119.10.1417 PMC1462955

[11] Dunaief JL (2011) Ironing out neurodegeneration: iron chelation for neuroprotection. Free Radic Biol Med 51: 1480–1481. 21616141 10.1016/j.freeradbiomed.2011.05.009 PMC4380125

[12] Komeima K, Rogers BS, Lu L, Campochiaro PA (2006) Antioxidants reduce cone cell death in a model of retinitis pigmentosa. Proc Natl Acad Sci U S A 103: 11300–11305. 16849425 10.1073/pnas.0604056103 PMC1544081

[13] Obolensky A, Berenshtein E, Lederman M, Bulvik B, Alper-Pinus R, et al (2011) Zinc-desferrioxamine attenuates retinal degeneration in the rd10 mouse model of retinitis pigmentosa. Free Radic Biol Med 51: 1482–1491. 21824515 10.1016/j.freeradbiomed.2011.07.014

[14] Yoshida N, Ikeda Y, Notomi S, Ishikawa K, Murakami Y, et al (2013) Laboratory evidence of sustained chronic inflammatory reaction in retinitis pigmentosa. Ophthalmology 120: e5–12. 10.1016/j.ophtha.2012.07.008 22986110

[15] Cingolani C, Rogers B, Lu L, Kachi S, Shen J, et al (2006) Retinal degeneration from oxidative damage. Free Radic Biol Med 40: 660–669. 16458197 10.1016/j.freeradbiomed.2005.09.032

[16] Chang B, Hawes NL, Pardue MT, German AM, Hurd RE, et al (2007) Two mouse retinal degenerations caused by missense mutations in the beta-subunit of rod cGMP phosphodiesterase gene. Vision Res 47: 624–633. 17267005 10.1016/j.visres.2006.11.020 PMC2562796

[17] Deleon E, Lederman M, Berenstein E, Meir T, Chevion M, et al (2009) Alteration in iron metabolism during retinal degeneration in rd10 mouse. Invest Ophthalmol Vis Sci 50: 1360–1365. 18997094 10.1167/iovs.08-1856

[18] Hahn P, Milam AH, Dunaief JL (2003) Maculas affected by age-related macular degeneration contain increased chelatable iron in the retinal pigment epithelium and Bruch’s membrane. Arch Ophthalmol 121: 1099–1105. 12912686 10.1001/archopht.121.8.1099

[19] Gargini C, Terzibasi E, Mazzoni F, Strettoi E (2007) Retinal organization in the retinal degeneration 10 (rd10) mutant mouse: a morphological and ERG study. J Comp Neurol 500: 222–238. 17111372 10.1002/cne.21144 PMC2590657

[20] Mori K, Duh E, Gehlbach P, Ando A, Takahashi K, et al (2001) Pigment epithelium-derived factor inhibits retinal and choroidal neovascularization. J Cell Physiol 188: 253–263. 11424092 10.1002/jcp.1114

[21] Obolensky A, Berenshtein E, Konijn AM, Banin E, Chevion M (2008) Ischemic preconditioning of the rat retina: protective role of ferritin. Free Radic Biol Med 44: 1286–1294. 18082149 10.1016/j.freeradbiomed.2007.10.060

[22] Meir T, Dror R, Yu X, Qian J, Simon I, et al (2007) Molecular characteristics of liver metastases from uveal melanoma. Invest Ophthalmol Vis Sci 48: 4890–4896. 17962435 10.1167/iovs.07-0215

[23] Collins TJ (2007) ImageJ for microscopy. Biotechniques 43: 25–30. 10.2144/000112517 17936939

[24] Vandesompele J, De Preter K, Pattyn F, Poppe B, Van Roy N, et al (2002) Accurate normalization of real-time quantitative RT-PCR data by geometric averaging of multiple internal control genes. Genome Biol 3: RESEARCH0034. 12184808 10.1186/gb-2002-3-7-research0034 PMC126239

[25] Livak KJ, Schmittgen TD (2001) Analysis of relative gene expression data using real-time quantitative PCR and the 2(-Delta Delta C(T)) Method. Methods 25: 402–408. 11846609 10.1006/meth.2001.1262

[26] Konijn AM, Levy R, Link G, Hershko C (1982) A rapid and sensitive ELISA for serum ferritin employing a fluorogenic substrate. J Immunol Methods 54: 297–307. 6816855 10.1016/0022-1759(82)90314-3

[27] Ohkawa H, Ohishi N, Yagi K (1979) Assay for lipid peroxides in animal tissues by thiobarbituric acid reaction. Anal Biochem 95: 351–358. 36810 10.1016/0003-2697(79)90738-3

[28] Reznick AZ, Cross CE, Hu ML, Suzuki YJ, Khwaja S, et al (1992) Modification of plasma proteins by cigarette smoke as measured by protein carbonyl formation. Biochem J 286 (Pt 2): 607–611. 10.1042/bj2860607 PMC1132941 1530591

[29] Reznick AZ, Packer L (1994) Oxidative damage to proteins: spectrophotometric method for carbonyl assay. Methods Enzymol 233: 357–363. 8015470 10.1016/s0076-6879(94)33041-7

[30] Chevion M, Berenshtein E, Stadtman ER (2000) Human studies related to protein oxidation: protein carbonyl content as a marker of damage. Free Radic Res 33 Suppl: S99–108 11191280

[31] Johansson LH, Borg LA (1988) A spectrophotometric method for determination of catalase activity in small tissue samples. Anal Biochem 174: 331–336. 3064653 10.1016/0003-2697(88)90554-4

[32] Chen M, Luo C, Penalva R, Xu H (2013) Paraquat-induced retinal degeneration is exaggerated in CX3CR1-deficient mice and is associated with increased retinal inflammation. Invest Ophthalmol Vis Sci 54: 682–690. 23299473 10.1167/iovs.12-10888

[33] Usui S, Komeima K, Lee SY, Jo YJ, Ueno S, et al (2009) Increased expression of catalase and superoxide dismutase 2 reduces cone cell death in retinitis pigmentosa. Mol Ther 17: 778–786. 19293779 10.1038/mt.2009.47 PMC2803613

[34] Usui S, Oveson BC, Iwase T, Lu L, Lee SY, et al (2011) Overexpression of SOD in retina: need for increase in H2O2-detoxifying enzyme in same cellular compartment. Free Radic Biol Med 51: 1347–1354. 21736939 10.1016/j.freeradbiomed.2011.06.010 PMC3163708

[35] Dong A, Shen J, Krause M, Akiyama H, Hackett SF, et al (2006) Superoxide dismutase 1 protects retinal cells from oxidative damage. J Cell Physiol 208: 516–526. 16741961 10.1002/jcp.20683

[36] He X, Hahn P, Iacovelli J, Wong R, King C, et al (2007) Iron homeostasis and toxicity in retinal degeneration. Prog Retin Eye Res 26: 649–673. 17921041 10.1016/j.preteyeres.2007.07.004 PMC2093950

[37] Chowers I, Wong R, Dentchev T, Farkas RH, Iacovelli J, et al (2006) The iron carrier transferrin is upregulated in retinas from patients with age-related macular degeneration. Invest Ophthalmol Vis Sci 47: 2135–2140. 16639025 10.1167/iovs.05-1135

[38] Banin E, Berenshtein E, Kitrossky N, Pe’er J, Chevion M (2000) Gallium-desferrioxamine protects the cat retina against injury after ischemia and reperfusion. Free Radic Biol Med 28: 315–323. 10699741 10.1016/s0891-5849(99)00227-0

[39] Chen H, Liu B, Lukas TJ, Suyeoka G, Wu G, et al (2009) Changes in iron-regulatory proteins in the aged rodent neural retina. Neurobiol Aging 30: 1865–1876. 18308429 10.1016/j.neurobiolaging.2008.01.002 PMC2789556

[40] Hadziahmetovic M, Kumar U, Song Y, Grieco S, Song D, et al (2012) Microarray analysis of murine retinal light damage reveals changes in iron regulatory, complement, and antioxidant genes in the neurosensory retina and isolated RPE. Invest Ophthalmol Vis Sci 53: 5231–5241. 22736611 10.1167/iovs.12-10204 PMC4159963

[41] Song D, Song Y, Hadziahmetovic M, Zhong Y, Dunaief JL (2012) Systemic administration of the iron chelator deferiprone protects against light-induced photoreceptor degeneration in the mouse retina. Free Radic Biol Med 53: 64–71. 22579919 10.1016/j.freeradbiomed.2012.04.020 PMC3380452

[42] Picard E, Jonet L, Sergeant C, Vesvres MH, Behar-Cohen F, et al (2010) Overexpressed or intraperitoneally injected human transferrin prevents photoreceptor degeneration in rd10 mice. Mol Vis 16: 2612–2625. 21179240 PMC3002967

