# Correction: Degeneration Modulates Retinal Response to Transient Exogenous Oxidative Injury

**DOI:** 10.1371/journal.pone.0099566

**Published:** 2014-05-30

**Authors:** The PLOS ONE Staff

The first two authors, Michal Lederman and Shira Hagbi-Levi, should be noted as contributing equally to this work.

## References

[1] Lederman M, Hagbi-Levi S, Grunin M, Obolensky A, Berenshtein E, et al (2014) Degeneration Modulates Retinal Response to Transient Exogenous Oxidative Injury. PLoS ONE 9(2): e87751 doi:10.1371/journal.pone.0087751 24586289 10.1371/journal.pone.0087751 PMC3931611

