# Supplementary figures and images for: Degeneration Modulates Retinal Response to Transient Exogenous Oxidative Injury
Source: PLoS One. 2014 Feb 21;9(2):e87751. doi: 10.1371/journal.pone.0087751 (PMC3931611; doi:10.1371/journal.pone.0087751)

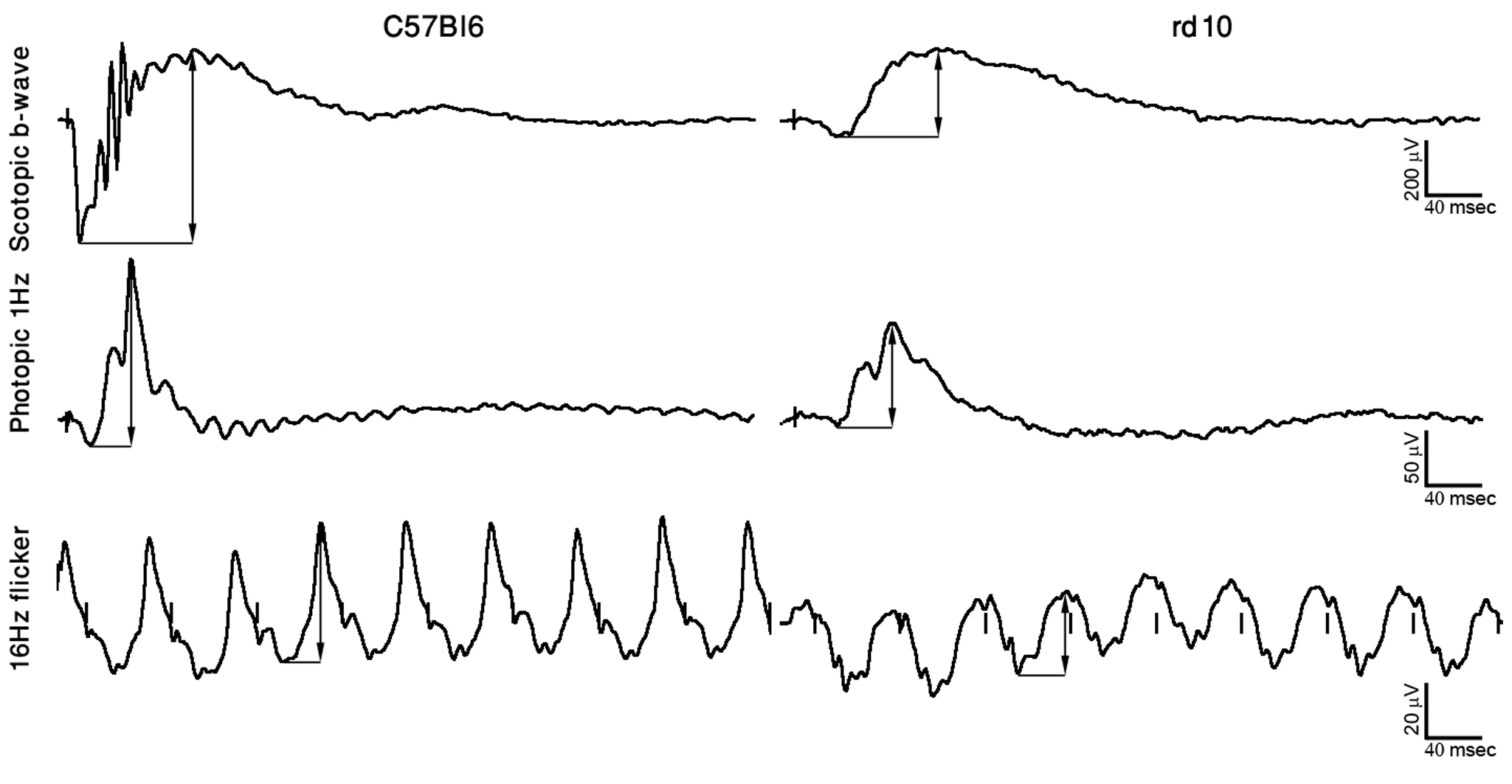

Supplement: Figure S1 — Traces of ERG recordings from WT (C57Bl6) and rd10 mice. The graph shows an example of measurements of b-wave response under scotopic and photopic conditions as well as measurements of 16 Hz flicker ERG response. Cursor position for measurement of the b-wave is indicated. (TIF) [file pone.0087751.s001.tif]
